# Synthetic data and ELSI-focused computational checklists—A survey of biomedical professionals’ views

**DOI:** 10.1371/journal.pdig.0000666

**Published:** 2024-11-20

**Authors:** Jennifer K. Wagner, Laura Y. Cabrera, Sara Gerke, Daniel Susser

**Affiliations:** 1 School of Engineering Design and Innovation, Penn State University, University Park, Pennsylvania, United States of America; 2 Department of Anthropology, Penn State University, University Park, Pennsylvania, United States of America; 3 Department of Biomedical Engineering, Penn State University, University Park, Pennsylvania, United States of America; 4 Institute for Computational and Data Sciences, Penn State University, University Park, Pennsylvania, United States of America; 5 Huck Institutes of the Life Sciences, Penn State University, University Park, Pennsylvania, United States of America; 6 Rock Ethics Institute, Penn State University, University Park, Pennsylvania, United States of America; 7 Penn State Law, University Park, Pennsylvania, United States of America; 8 Department of Engineering Science and Mechanics, Penn State University University Park, Pennsylvania, United States of America; 9 Department of Philosophy, Penn State University, University Park, Pennsylvania, United States of America; 10 Bioethics Program, Penn State University, University Park, Pennsylvania, United States of America; 11 Penn State Dickinson Law, Carlisle, Pennsylvania, United States of America; 12 University of Illinois Urbana-Champaign, College of Law, Champaign, Illinois, United States of America; 13 Department of Information Science, Cornell University, Ithaca, New York, United States of America; Yonsei University College of Medicine, REPUBLIC OF KOREA

## Abstract

Artificial intelligence (AI) and machine learning (ML) tools are now proliferating in biomedical contexts, and there is no sign this will slow down any time soon. AI/ML and related technologies promise to improve scientific understanding of health and disease and have the potential to spur the development of innovative and effective diagnostics, treatments, cures, and medical technologies. Concerns about AI/ML are prominent, but attention to two specific aspects of AI/ML have so far received little research attention: synthetic data and computational checklists that might promote not only the reproducibility of AI/ML tools but also increased attention to ethical, legal, and social implications (ELSI) of AI/ML tools. We administered a targeted survey to explore these two items among biomedical professionals in the United States. Our survey findings suggest that there is a gap in familiarity with both synthetic data and computational checklists among AI/ML users and developers and those in ethics-related positions who might be tasked with ensuring the proper use or oversight of AI/ML tools. The findings from this survey study underscore the need for additional ELSI research on synthetic data and computational checklists to inform escalating efforts, including the establishment of laws and policies, to ensure safe, effective, and ethical use of AI in health settings.

## Author summary

Many efforts are underway to promote ethical and trustworthy AI/ML for biomedical research and care, including the BRIDGE2AI initiative supported by the National Institutes of Health (NIH). We conducted an exploratory survey of biomedical professionals in the United States to guage their perspectives on two specific matters that had received little attention to date: synthetic data and checklists focused on ethical, legal, and social implications (ELSI) of AI/ML. We found a preference for actual data over synthetic data and general interest in the possible utility of ELSI-focused computational checklists to increase attention given to important matters (such as addressing bias, preserving privacy, and promoting transparency of AI/ML tools). Our survey findings highlight the need for increased awareness among biomedical professionals on these matters. They also underscore the importance for more ELSI research on AI/ML to inform the growing policy efforts underway within the United States and around the world to ensure that biomedical uses of AI are safe, effective, ethical, and trustworthy.

## Introduction

Artificial intelligence (AI), machine learning (ML), and related technological advances in data sciences are disrupting society generally and healthcare specifically at a dizzying pace. Regulatory agencies, including the Food and Drug Administration (FDA), have been exploring and developing approaches to AI/ML oversight within existing medical device regulatory frameworks for several years [e.g, 1–8]. However, with the issuance of the Blueprint for an AI Bill of Rights in October 2022 by the White House Office of Science and Technology Policy [9] and with the Executive Order on AI signed by President Biden in October 2023 [10], administrative agencies and private entities are jumping to action to accelerate AI for biomedical purposes while trying to ensure the technologies are developed and deployed responsibly and ethically. The Executive Order emphasizes that potential harms caused or exacerbated by AI including (but not limited to) privacy, bias, and discrimination must be mitigated in healthcare contexts. Among the many mandates of the Executive Order is the establishment of an “HHS AI Task Force” to create, within the next year, a strategic plan—along with policies, frameworks, appropriate regulatory action, guidance, and resources—for AI and AI-enabled technologies in the health sector. The Executive Order further requires the Secretary of Health and Human Services to develop an AI assurance policy, to take action to ensure appropriate understanding of and compliance with federal nondiscrimination laws, establish an AI safety program, develop a regulatory strategy for the use of AI in drug development, and continue research funding for AI-related work (such as the AIM-AHEAD program [11]).

One dimension of the AI/ML story that deserves more attention is the use of synthetic data (SD), which has been of increasing interest for precision medicine with several distinct use cases [12]. SD has been characterized as having particular value in biomedical science because it could help overcome institutional and regulatory obstacles to sharing actual patient data, preserve privacy, and reduce “time to insights” [13]. There is no consensus definition of SD [e.g., 14], but one working definition describes SD as “data that has been generated using a purpose-built mathematical model or algorithm, with the aim of solving a (set of) data science task(s)” [15]. There has been little empirical research on how health scientists and practitioners understand the meaning, potential, and limitations of synthetic data; however, given the “high stakes” nature of healthcare applications,[14] making sure everyone—whether biomedical AI/ML users (e.g., scientistists and clinicians) or those responsible for ensuring adequate patient-participant protections are in place (e.g., ethicists)—is on the same page is critical. Communication and explanation of SD is important both prior to the use of SD (e.g., within informed consent processes) and afterwards when reporting results (e.g., research findings or healthcare outcomes), and many audiences and their corresponding knowledge gaps and perspectives of SD must be considered (including, e.g., academic scholars, journalists, corporate executives, policymakers, patients, communities, trainees/students, and the general public). Misunderstandings could lead to both unjustified reliance on or rejection of SD, impeding the realization of its benefits or risking avoidable harm.

Addressing SD-related ethical, legal, and social issues is challenging, at least in part because of the definitional problems, as some scholars have already begun noting SD sits in a “regulatory blind spot” [14]. As with AI/ML more broadly, there are many advocates for soft law or collaborative governance approaches [e.g., 16] that leverage existing laws along with professional norms and who call on professional societies and members of the technology industry to help set expectations for conduct. Recently, leading members of the technology industry announced their willingness to adhere to voluntary guidelines for AI/ML [17] and even called for legislative reform [e.g., [18]] to bring clarity where there is current legal and regulatory uncertainty over responsibilities. Benchmarking [e.g., [19]] and computational checklists have been viewed as an attractive option to encourage data scientists developing, improving, and using AI/ML models to approach their work thoughtfully and to disclose metadata that could, among other desirable purposes, facilitate reproducibility. Integrating components of ELSI (i.e., ethical, legal, and social issues) into AI/ML computational checklists has been suggested as having potential benefits [20–22]. But what issues ELSI-focused computational checklists might reasonably address, how they might work in practice, and what might impede or facilitate their adoption has not received much research attention.

To help uncover ways in which ethical and trustworthy biomedical AI/ML could be advanced (within and beyond the BRIDGE2AI initiative, an NIH Common Fund program intended to address these complex issues and develop best practices [23]), we undertook a suite of complementary efforts, including qualitative interview research and convening a national academic work group. Here, we report on the exploratory survey research we conducted to surface perspectives among biomedical professionals about synthetic data and ELSI-focused computational checklists.

## Methods

A survey instrument was developed and refined based on preliminary findings from key informant interviews (which are under review elsewhere) before being programmed into Qualtrics (Qualtrics LLC; Provo, UT, USA) for online administration. The integration of Tango Rewards Genius enabled delivery of a $25.00 research incentive for completion of the survey to those respondents who wished to receive it. The survey instrument (S1 Appendix) included an information page before survey participants could proceed to the 30 questions of the survey, which consisted of a series of questions about the participants and substantive questions about their perspectives regarding (a) synthetic data (which was not defined in the instrument), (b) ethics-focused computational checklists for AI/ML, and (c) AI in society generally. The survey included both closed and open questions. The activities involved in this study (“Exploring the Ethics of Synthetic Data and Artificial Intelligence,” Study00020918) were determined to be exempt research by the Institutional Review Board at Penn State University on August 30, 2022.

A survey recruitment pool was created by identifying adults in the United States with relevant professional expertise who appear to be employed by an organizational member of the Association of American Medical Colleges (AAMC) [24–25]. Relevant expertise in AI or AI-related ethics was determined based on employment in a relevant role (based on title or description) suggesting that the individual might be an AI/ML developer or user (such as a data scientist, data engineer, informaticist, chief information officer, etc.) or might be involved with AI/ML ethics, policy, or oversight (such as an ethicist, institutional review board member, chief bioethics officer, ethics researcher, etc.). A random sampling of the 544 organizational members of the AAMC (comprising hospital/health systems and medical schools) was taken, and, from those AAMC member organizations, six (6) employees (comprising approximately an even distribution of roles, AI and AI ethics) were identified from publicly accessible information (such as employee directories and other organizational webpages, press releases, published literature, etc.). A modified Dillman approach [26] was used to administer the survey in which individuals in the recruitment pool were contacted directly by the study team using a series of three email messages: (1) a pre-survey message sent in advance to notify individuals of the forthcoming survey, (2) a message with a URL to the online survey, and (3) a reminder message that again included a URL to the online survey. Initial recruitment messages were sent to N = 771 individuals across 146 organizations. There were N = 49 instances of failed email delivery. Survey responses were collected between June 16, 2023 and August 28, 2023.

Our goal was to collect a sample of N = 250 responses to have sufficient power to perform simple comparisons (e.g., comparisons between subgroups of n = 50 would provide 80% statistical power at alpha = 0.05 to detect relatively small effect sizes of 0.23). Ultimately, however, we were performing this survey for a modest purpose: to augment the perspectives of the members of the academic working group (consisting of eight scholars in addition to the study team) convened to explore these issues. Thus, even a small sample size would have data adequacy [e.g., [27–28]] for purposes of issue spotting and generating hypotheses appropriate for subsequent AI ethics research. Microsoft Excel was used to calculate descriptive statistics and conduct Chi-square tests of independence. Chi-square tests of independence were performed only for a subset of questions to assess statistically significant differences in responses among AI developers/users and AI ethicists. For those analyses, the respondents who did not self-categorize into either group were excluded.

## Results

A total of 82 adults in the United States with relevant expertise responded to our survey; however, seven (7) of the participants failed to respond to any of the substantive questions, and those survey responses were excluded from analysis. Thus, the survey sample for analysis consisted of responses from 75 adults in the United States with relevant expertise, reflecting an overall survey response rate of 10.4% (75/722). Three (3) respondents completed the questions about themselves and synthetic data but then abandoned the survey before the questions pertaining to computational checklists and AI generally. Those were retained for analysis. The mean time to completion of the survey was 18.9 minutes. A descriptive summary of our survey participants’ characteristics is displayed in Table 1. While the sample is diverse with regard to age, gender identity, workplace type, professional role, years of professional experience, and geographic region, the sample consists predominantly of individuals who reported being highly educated (96% reporting an advanced degree) and White (71.2%).

**Table 1:** Descriptive Summary of Survey Respondents.

	Count* (N)	Frequency (%)
**Age**		
18–25 years	2	2.7
26–35 years	13	17.3
36–45 years	24	32.0
46–55 years	17	22.7
56–65 years	14	18.7
66–75 years	5	6.7
**Gender Identity**		
Male	34	45.3
Female	40	53.3
Non-binary	1	1.3
**Race/Ethnicity**		
Asian	12	16.4
Hispanic, Latino, or Spanish	2	2.7
White	52	71.2
More than one selection	7	9.6
**Educational Attainment**		
College 4 years or more (college graduate)	3	4.0
Advanced degree (master’s, doctorate, etc.)	73	96.0
**Workplace**		
Medical School	25	35.2
Teaching Hospital or Healthcare System	26	36.6
College or University	20	28.2
**Professional Role**		
AI/ML developer or user (or similar)	24	32.0
Ethicist (or similar)	27	32.0
Other**	24	32.0
**Experience in Professional Role**		
0–5 years	23	30.7
6–10 years	8	10.7
More than 10 years	44	58.7
**Geographic Region** ***		
Northeast	20	27.4
South	21	28.8
Midwest	23	31.5
West	9	12.3

*Totals for each item do not necessarily sum to N = 75 for all respondents due to item nonresponse or selections of options that are not displayed.

**Individuals who declined to self-categorize into one of the two provided options overwhelmingly (18/24) offered information suggesting they should be categorized among the AI/ML developers or users (e.g., “Physician, ai/ml user”). Others offered ambiguous descriptions (e.g., “professor” or “researcher” without any indication of discipline or type of expertise). Nevertheless, these respondents have been retained as a separate group for descriptive purposes.

***Geographic region relies upon the groupings of states per U.S. Census Bureau as Northeast (CT, ME, MA, NH, NJ, NY, PA, RI, VT); Midwest (IL, IN, IA, KS, MI, MN, MO, NE, ND, OH, SD, WI); South (AL, AR, DE, DC, FL, GA, KY, LA, MD, MS, NC, OK, SC, TN, TX, VA, WV); and West (AK, AZ, CA, CO, HI, ID, MT, NM, NV, OR, UT, WA, WY).

### Familiarity with Subject Matter

**Fig 1:**
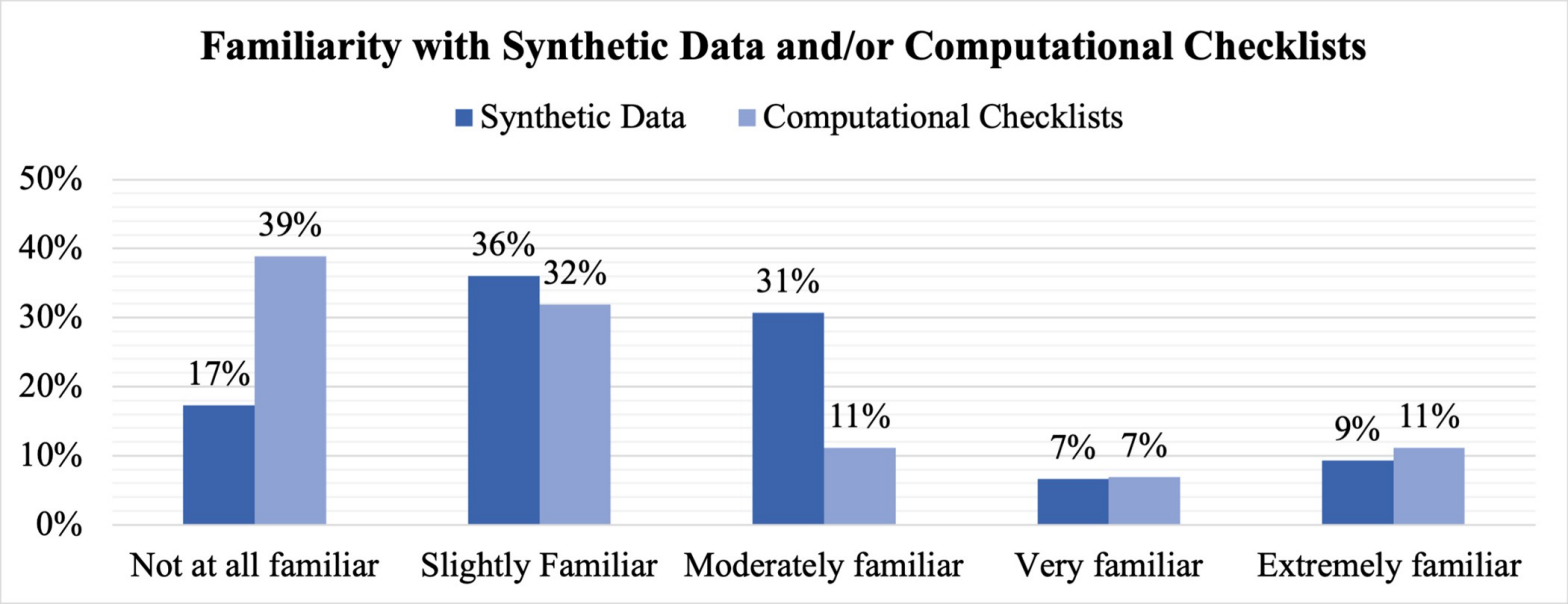
Familiarity with Synthetic Data and/or Computational Checklists. Illustrated is the reported familiarity with these items for the entire survey sample (N = 75). Dark blue shading refers to familiarity with synthetic data, and light blue shading refers to familiarity with computational checklists.

**Fig 2:**
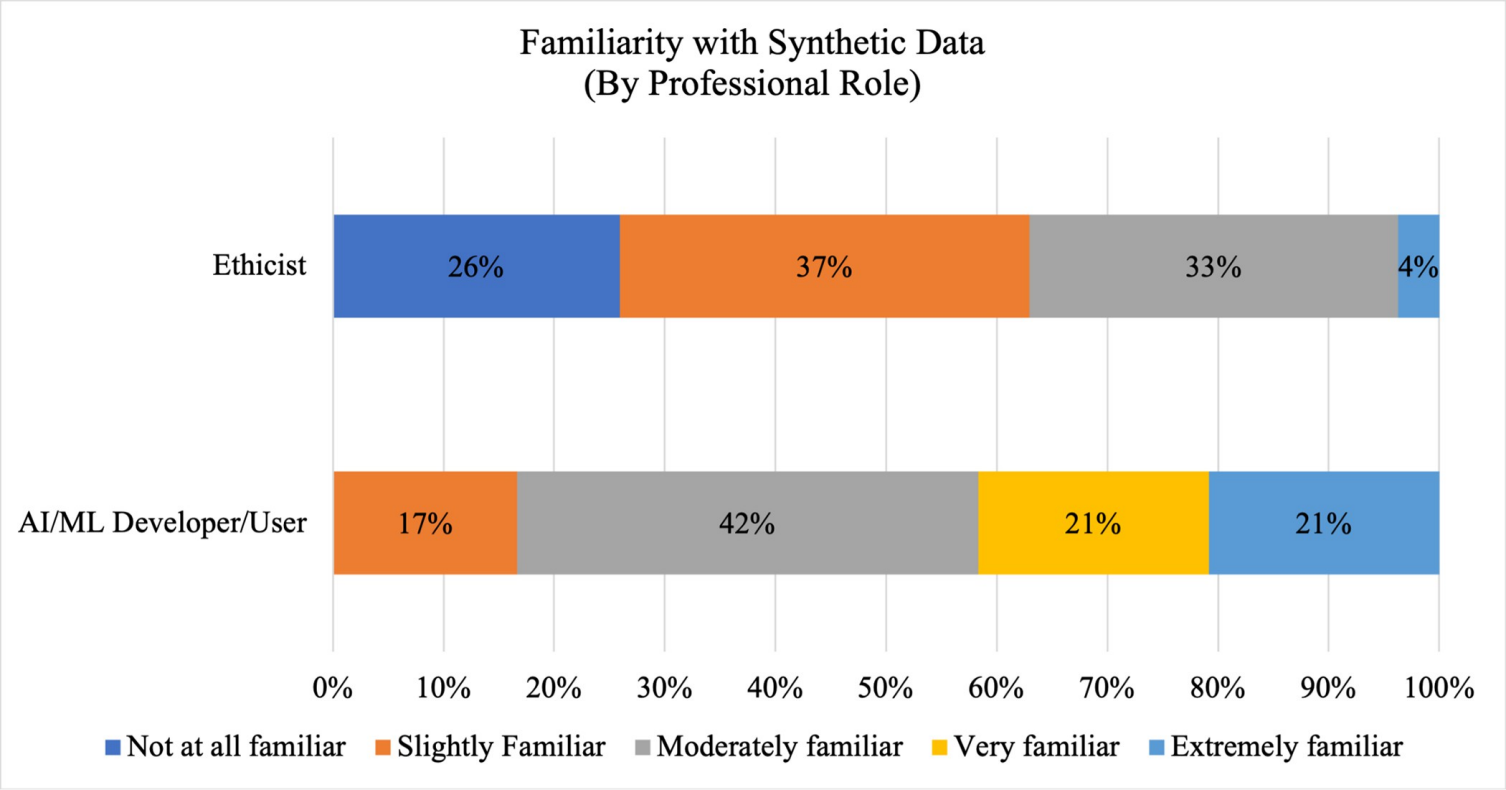
Familiarity with Synthetic Data By Professional Role. A comparison of familiarity among AI/ML developers/users (N = 24) and AI/ML-related ethicists (N = 27) with synthetic data is shown. Dark blue shading indicates no familiarity; orange shading indicates slight familiarity; grey shading indicates moderate familiarity; yellow shading indicates very familiar responses; and light blue shading indicates extreme familiarity.

**Fig 3:**
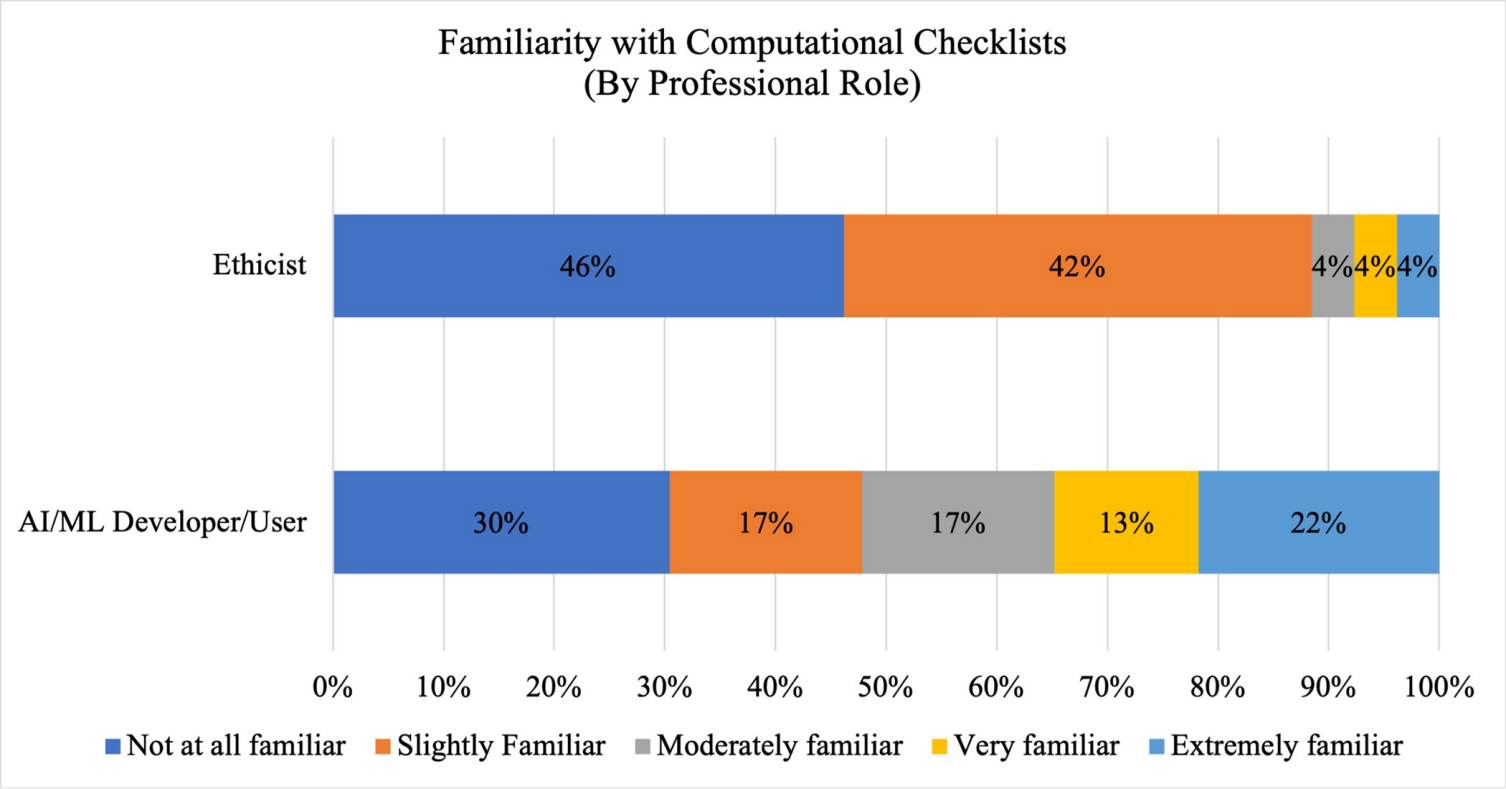
Familiarity with Computational Checklists By Professional Role. A comparison of familiarity among AI/ML developers/users (N = 23) and AI/ML-related ethicists (N = 26) with computational checklists is shown. Dark blue shading indicates no familiarity; orange shading indicates slight familiarity; grey shading indicates moderate familiarity; yellow shading indicates very familiar responses; and light blue shading indicates extreme familiarity.

Despite being employed at institutions and in professional roles in which we could reasonably expect individuals to encounter synthetic data and computational checklists, respondents indicated limited familiarity with either. As shown in Fig 1, a majority of respondents (53.3%, 40/75) indicated they were either not at all or slightly familiar with synthetic data, and only 16.0% (12/75) indicated they were very or extremely familiar with synthetic data. The remainder (30.7%, 23/75) indicated they were moderately familiar with synthetic data. Similarly, respondents were overwhelmingly unfamiliar with computational checklists, as 70.8% (51/72) of respondents indicated they were either not at all or slightly familiar, 11% (8/72) of respondents indicated they were moderately familiar, and 18.1% (13/72) of respondents indicated they were either very or extremely familiar with computational checklists. As shown in Figs 2 and 3, familiarity with synthetic data and computational checklists varies by professional role. While one-quarter of respondents with an ethics-related role (26%, 7/27) expressed they were not at all familiar with synthetic data, all respondents with an AI/ML developer or user role were at least slightly familiar with synthetic data, and more than 40% (41.6%, 10/24) reported being either very or extremely familiar with synthetic data. A null hypothesis of independence of professional role and familiarity with synthetic data was rejected at α = 0.05 (χ^2^ = 17.2; df = 4; p-value = 0.00179), and a similar null hypothesis of independence of professional role and familiarity with computational checklists was rejected at α = 0.05 (χ^2^ = 11.0; df = 4; p-value = 0.02622).

### Results Specifically Regarding Synthetic Data

**Fig 4:**
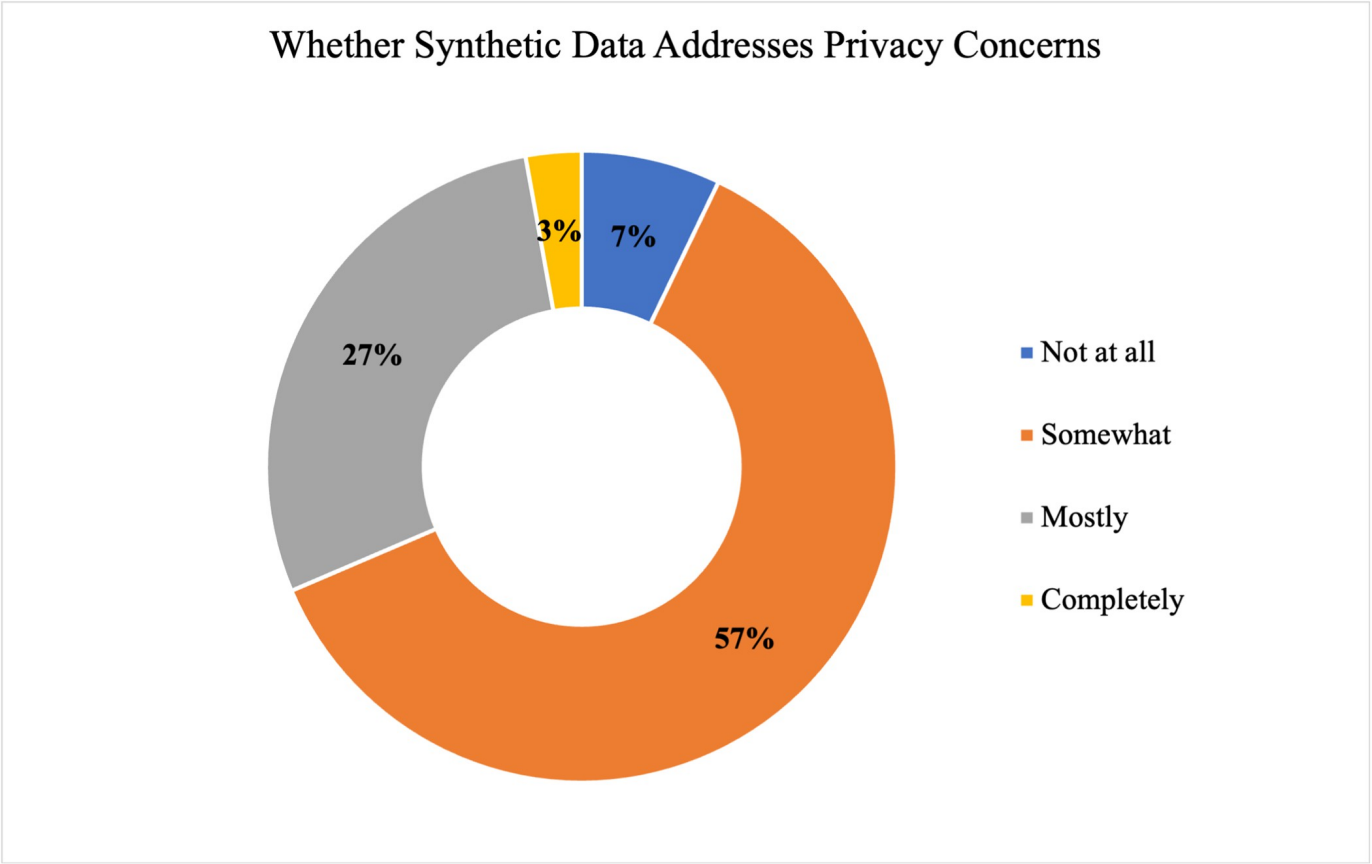
Perspectives Regarding Whether Synthetic Data Addresses Privacy Concerns. Perspectives of all respondents (N = 75) on the extent to which a possible benefit of SD is that it is able to address privacy concerns are shown, with the perspective “not at all” in dark blue, “somewhat” in orange, “mostly” in grey, and “completely” in yellow.

**Fig 5:**
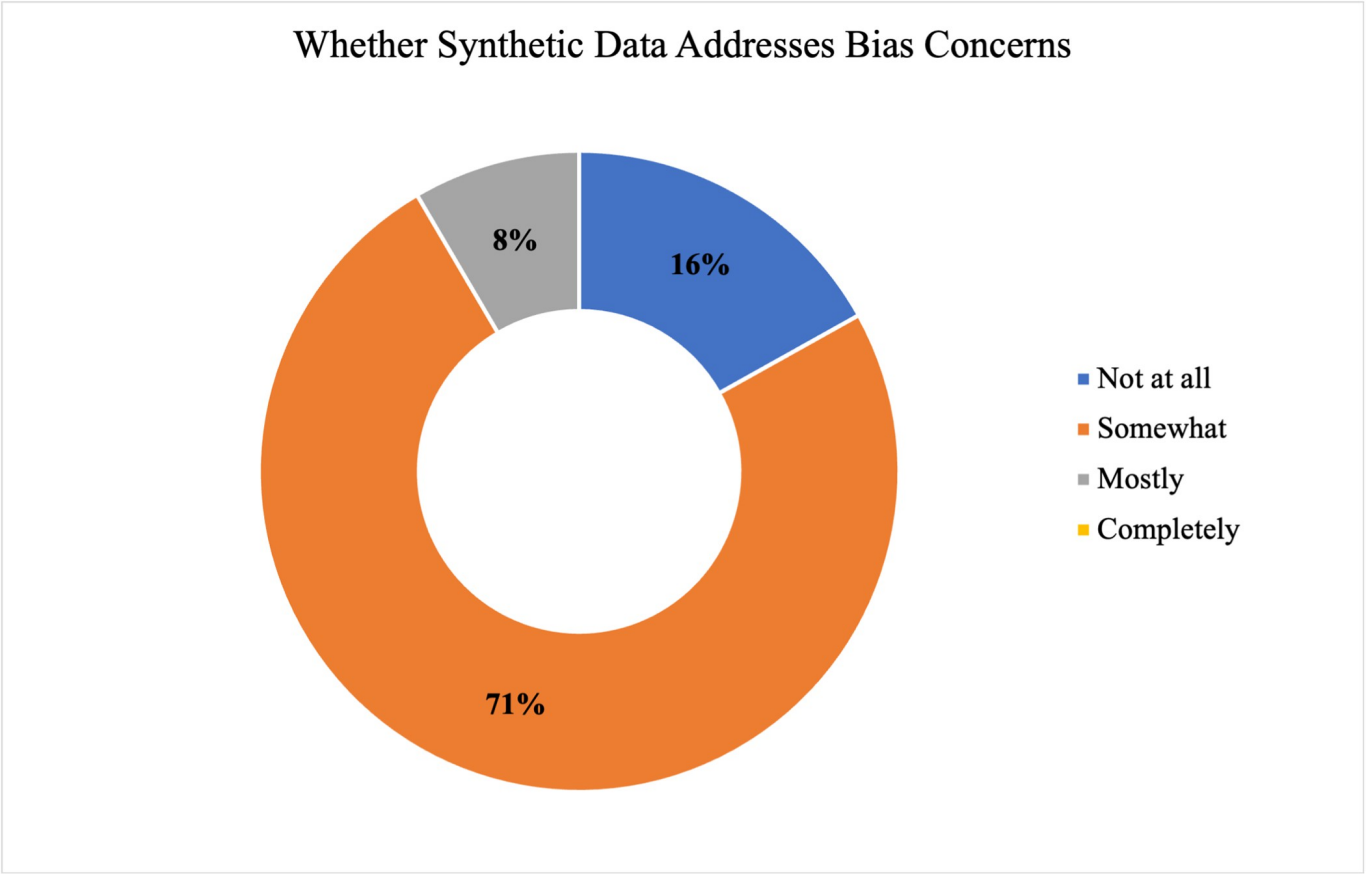
Perspectives Regarding Whether Synthetic Data Addresses Bias Concerns. Perspectives of all respondents (N = 75) on the extent to which a possible benefit of SD is that it is able to address bias are shown, with the perspective “not at all” in dark blue, “somewhat” in orange, “mostly” in grey, and “completely” in yellow.

A majority of respondents (57% or 43/75) indicated they had neither a favorable nor unfavorable opinion of synthetic data. A null hypothesis of independence of professional role and (un)favorable opinion of synthetic data was not rejected at α = 0.05 (χ^2^ = 3.89; df = 4; p-value = 0.42134). Two-thirds of respondents (66.7%, 50/75) indicated that they generally preferred actual, real data over synthetic data, only one respondent indicated a general preference for synthetic data, and the remainder (32%, 24/75) reported no preference. Regarding possible benefits of synthetic data (Figs 4 and 5), a majority of responses suggested that synthetic data could “somewhat” address health information privacy concerns (57.3%, 43/75) and bias concerns (70.7%, 53/75).

**Fig 6:**
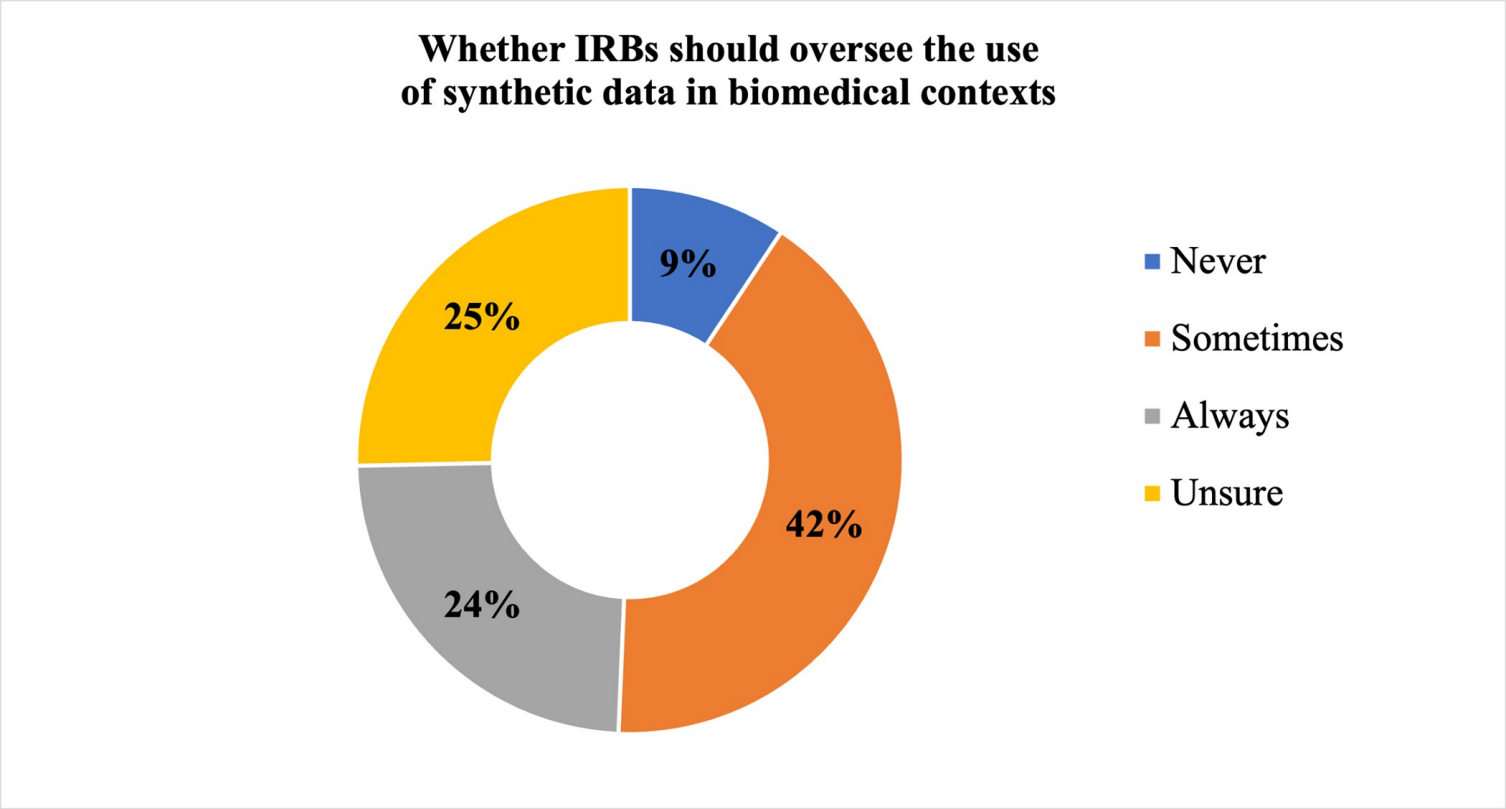
Perspectives Regarding Whether IRBs Should Oversee the Use of Synthetic Data in Biomedical Contexts. Perspectives of all respondents (N = 75) on the extent to which IRBs should be involved in oversight of biomedical uses of SD are shown, with the perspective “never” in dark blue, “sometimes” in orange, “always” in grey, and “unsure” in yellow.

Respondents were divided on whether an institutional review board (IRB) should oversee use of synthetic data in biomedical contexts (Fig 6), with 9.3% (7/75) indicating IRBs never should be involved, 24.0% (18/75) indicating IRBs always should be involved, 41.3% (31/75) indicating IRBs sometimes should be involved, and 25.3% (19/75) being unsure. A null hypothesis of independence of professional role and perspectives about IRB oversight of synthetic data was not rejected at α = 0.05 (χ^2^ = 0.49; df = 3; p-value = 0.92044). Respondents who elaborated on the reasons for their perspective regarding IRB oversight were diverse, with several questioning whether IRBs had the appropriate expertise or authority. Several remarked that synthetic data “doesn’t belong to a human subject”; are not “about human subjects”; or are “not real data” and, as such, indicated the IRB lacks authority. Some noted oversight might be important even if research with synthetic data is exempt from Common Rule regulation (e.g., “because of the downstream implications for humans”), while others were concerned about IRB overreach. Yet other respondents indicated that IRBs could have a role in the generation of synthetic data but not in their uses. A few noted whether IRB oversight is appropriate depends upon the method used for generating the synthetic data and whether synthetic data are the only data involved in the activity.

Of those who expressed an oversight role for the IRB, points raised included “the integrity of science,” the validity of the synthetic data and avoiding “manipulation of data,” whether proper steps to address bias have been taken, privacy implications for individuals and groups (such as inadvertent representation or disclosure of protected health information), and appropriate presentation of findings to avoid misleading others. For example, one respondent (who was a self-described biomedical scientist with more than 10 years of experience) noted, “IRB’s [sic] are in a unique position to flag inconsistencies with real world data and, in my opinion, would be the entity with enough authority to do something about erroneous data” while another respondent (who self-categorized as an ethicist with more than 10 years of experience) explained, “I honestly do not know exactly what ‘synthetic data’ are. Sounds like research fraud to me!” While some respondents expressed a protectionist view that it is better to err on the side of unnecessary oversight for emerging technologies (e.g., “…safer to have the IRB overseeing any field involving new techs such as synthetic data”), others expressed opposition to perceived unnecessary interference of IRBs (e.g., “Synthetic data broadens access by AI/ML developers. IRB oversight creates additional barriers for AI/ML developers.”). Additionally, two respondents who both supported a role for IRBs explained as follows:

“Because human eyes have to look at it. We are not all robots yet.”

“Because there should be someone who looks at what is exactly the PI and the research team trying to do in terms of Human research. The term ‘synthetic data’ is too loosely utilized and how are the ‘synthetic data’ produced can border inferring wrong conclusions based on artificially created data or research misconduct if the wrong data fields are ‘synthetically’ produced and then utilized in the analyses. It will not be easy for the IRB boards to identify the specialists who can look carefully at the proposals that include these ‘synthetic data’ proposals. Also very specific guidelines have to be given to the PIs when they prepare proposals that utilize these techniques.”

Most respondents indicated the quality of synthetic data might be difficult to determine (82.7%, 62/75), and a majority of respondents indicated researchers using synthetic data might not disclose that their studies rely on synthetic data (65.3%, 49/75), synthetic data might exacerbate data inequities (65.3%, 49/75), and that synthetic data might disincentivize researchers from engaging individuals, groups, and communities who are underrepresented (50.7%, 38/75). A sizable minority of respondents also indicated synthetic data might cause problems with accountability (45.3%, 34/75), synthetic data might be used to evade human subjects research protections (42.7%, 32/75), and synthetic data uses might disincentivize researchers from returning study findings to individuals, groups, and communities (42.7%, 32/43).

### Results Specifically Regarding ELSI-focused computational checklists

**Fig 7:**
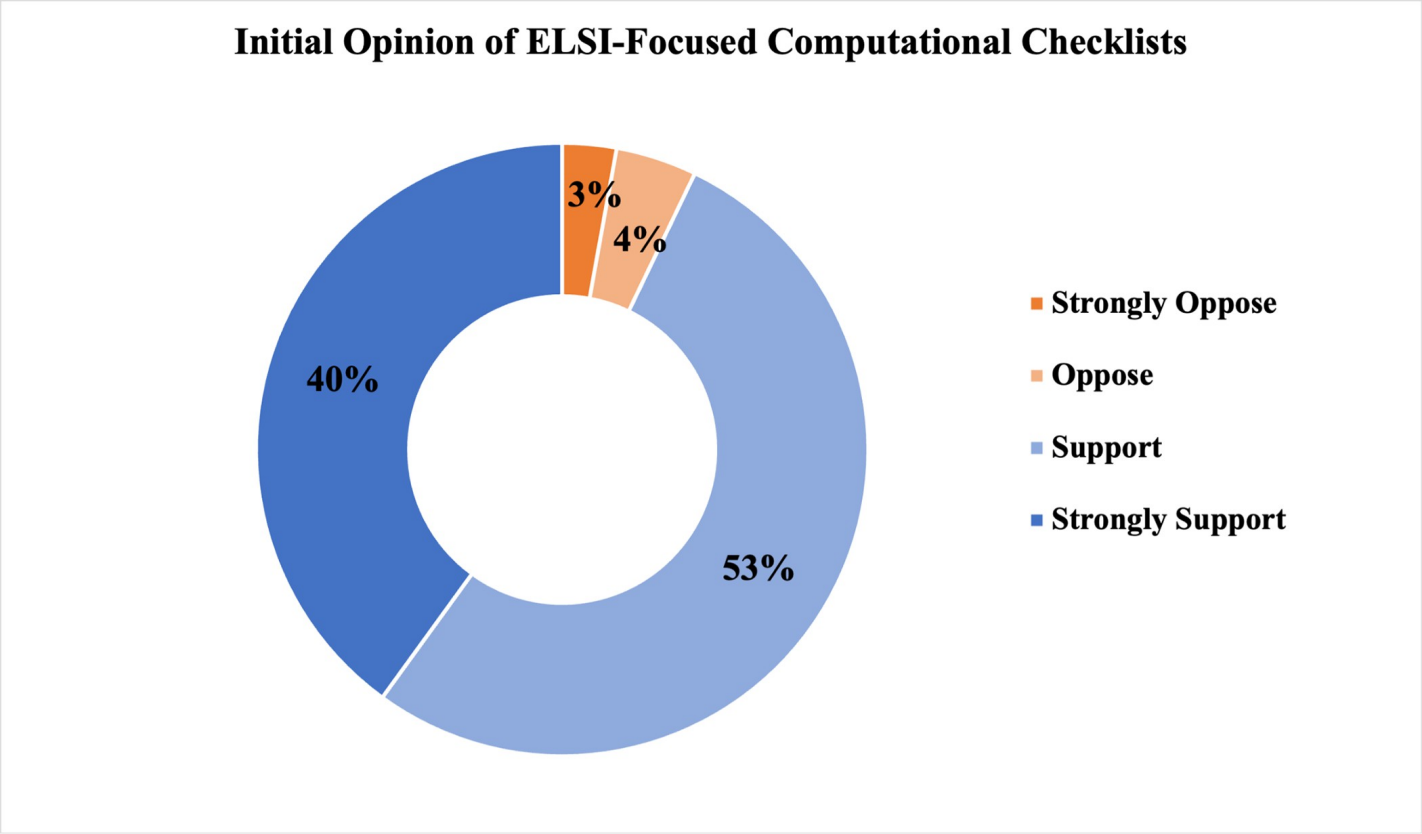
Initial Opinion of ELSI-Focused Computational Checklists. Support for and opposition to the notion of ELSI-focused computational checklists among respondents (N = 70) is shown, with support and strong support displayed in increasing shades of blue and opposition and strong opposition in increasing shades of orange.

**Fig 8:**
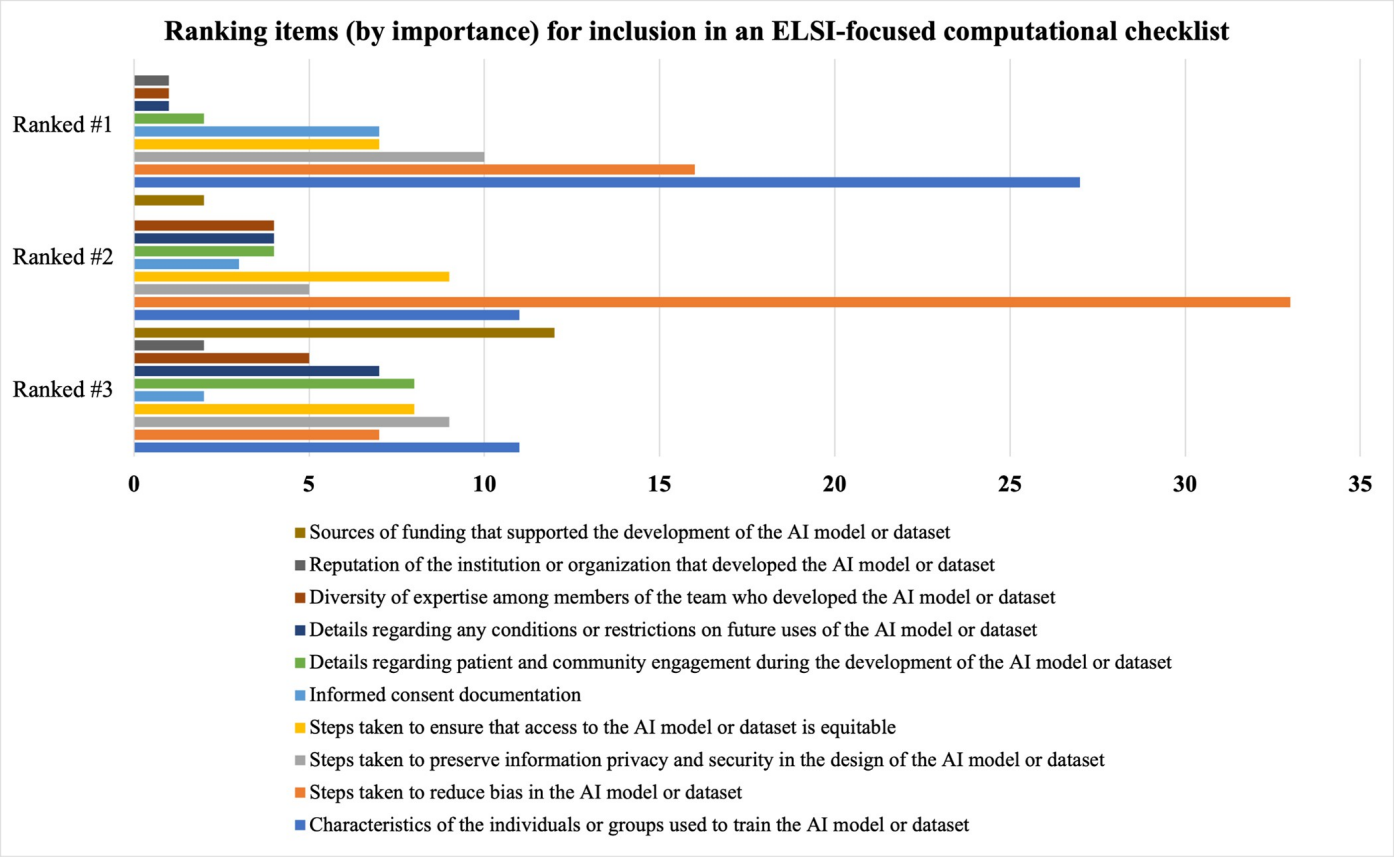
Ranking Items (by Importance) for Inclusion in an ELSI-Focused Computational Checklist. Respondents’ (N = 75) prioritization of the top three items for inclusion in an ELSI-focused computational checklists are shown. Each item appears as a different color. Steps taken to reduce bias and characteristics of those whose data were used to train the AI model or dataset were most ranked in the top three, followed by a tie among steps taken to preserve information privacy and security in the design of the AI model or dataset and steps taken to ensure that access to the AI model or dataset is equitable.

Respondents were overwhelmingly supportive of the idea of creating and using computational checklists for ethics-related aspects of AI models and data sets, with 92.8% of respondents (65/70) indicating their support (52.9%, 37/70) or strong support (40.0%, 28/70) as shown in Fig 7. From a prepopulated list of eight items, respondents ranked their top three items by importance for inclusion in an ELSI-focused computational checklist, as shown in Fig 8. The item most commonly ranked in the top three (whether in the #1, #2, or #3 ranking) was steps taken to reduce bias in the AI model or dataset (74.7%, 56/75) followed by characteristics of the individuals or groups used to train the AI model or dataset (65.3%, 49/75) and also by a tie (32.0%, 24/75, mean rank) between steps taken to preserve information privacy and security in the design of the AI model or dataset and steps be taken to ensure that access to the AI model or dataset is equitable.

**Fig 9:**
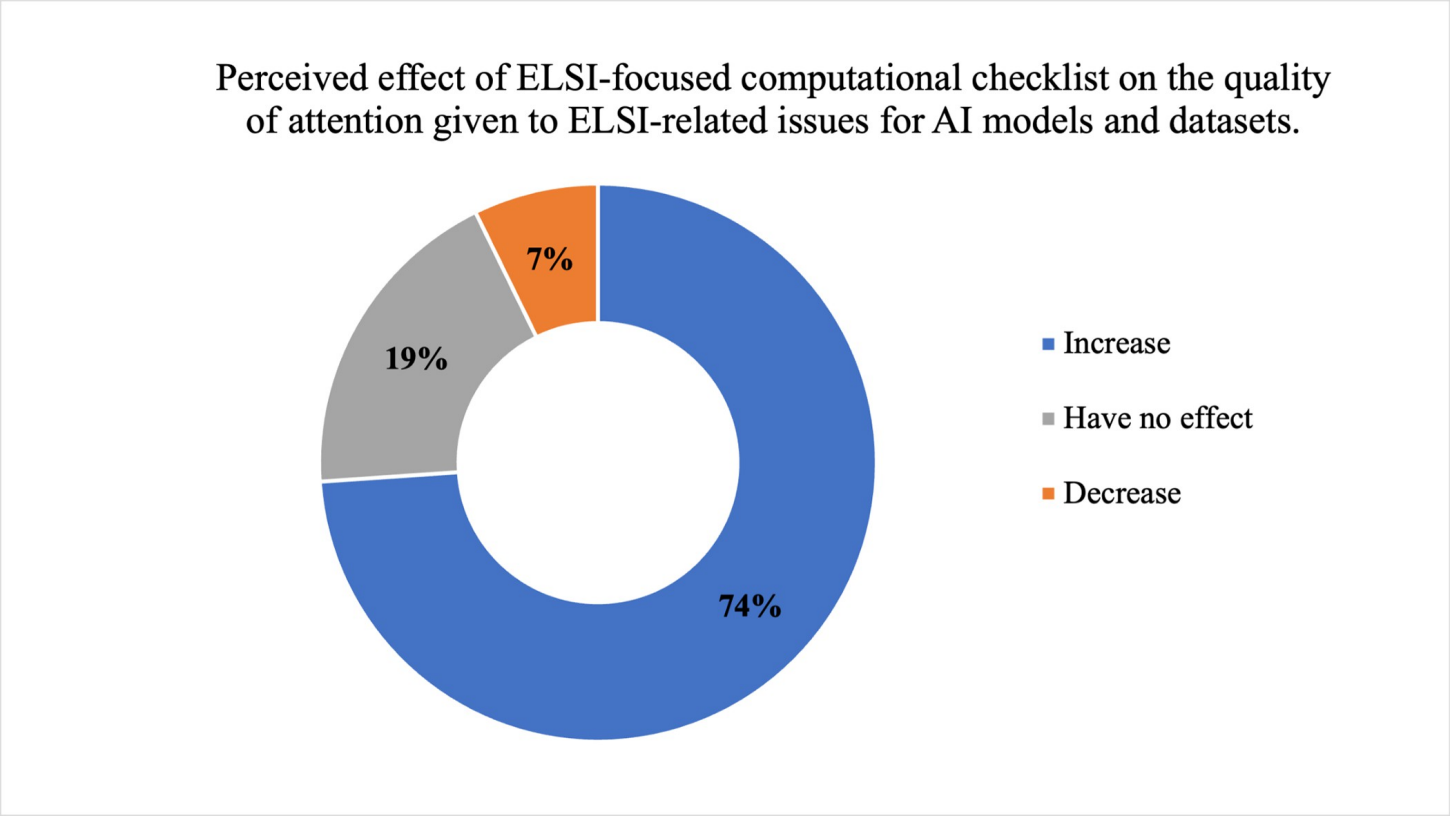
Perceived Effect of ELSI-Focused Computational Checklist on the Quality of Attention Given to ELSI-related Issues for AI models and datasets. Respondents’ (N = 69) perception that an ELSI-focused computational checklist would increase the quality of attention given to those matters appears in dark blue, that it would have no effect appears in grey, and that it would decrease the quality of attention appears in dark orange.

**Fig 10:**
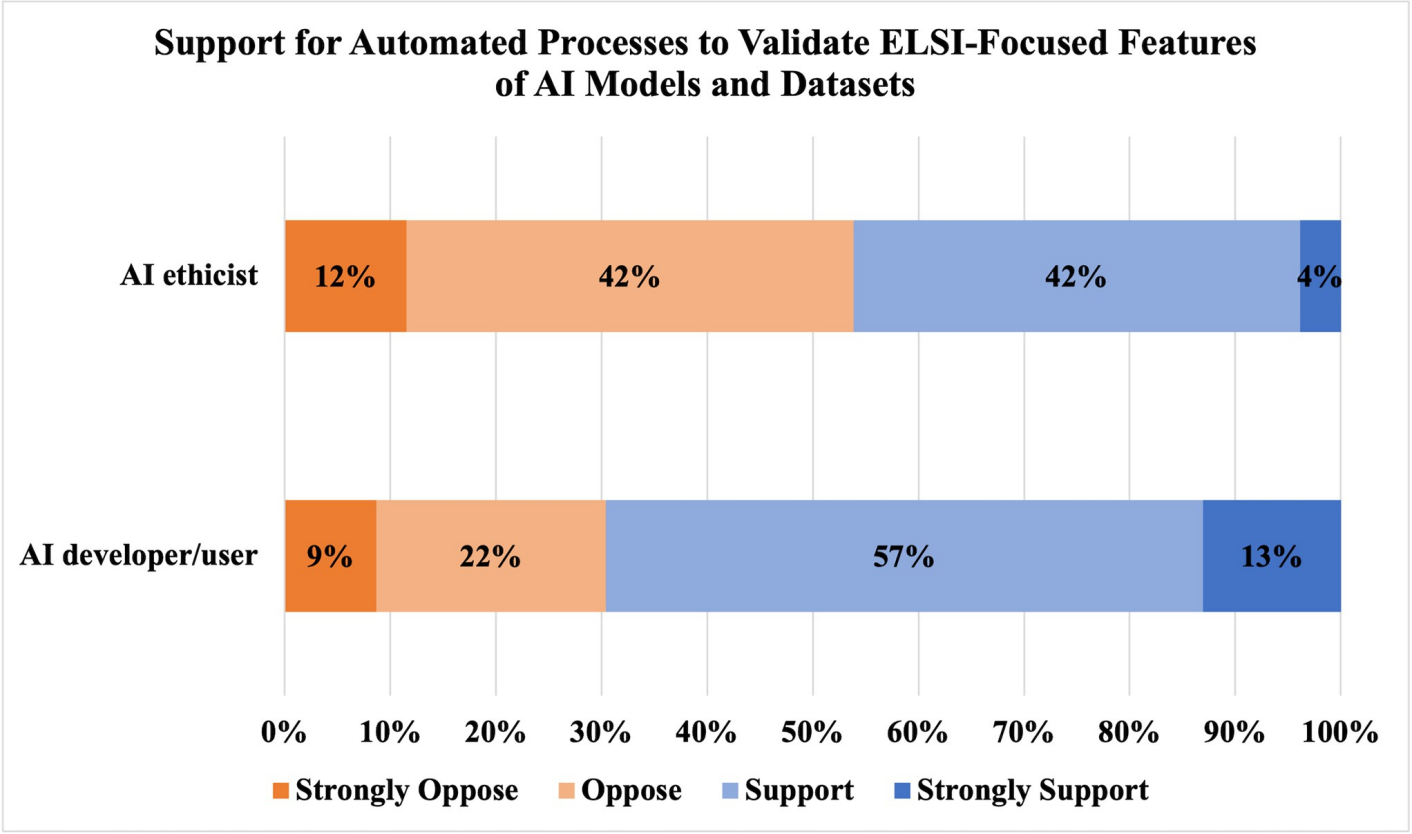
Support for Automated Processes to Validate ELSI-Focused Features of AI Models and Datasets. A comparison of respondents’ (N = 49) support for and opposition to automated processes to validate ELSI-focused features among AI/ML developers/users (N = 23) and AI/ML-related ethicists (N = 26) is displayed, with support and strong support displayed in increasing shades of blue and opposition and strong opposition in increasing shades of orange.

Respondents’ perceptions are generally that ethics-focused computational checklists would increase the quality of attention given to ethical dimensions of AI models and datasets (73.9%, 51/69), as shown in Fig 9. A majority of respondents (55.7%, 39/70) expressed support (48.6%, 34/70) or strong support (7.1%, 5/70) for the idea of automated processes to validate disclosures of ethics-focused features of AI models and datasets. While support for such automated validation processes appeared to vary by professional role, as shown in Fig 10, a null hypothesis of independence of professional role and support for automated processes to validate disclosures of ethics-focused features of AI models and datasets was not rejected at α = 0.05 (χ^2^ = 2.73; df = 1; p-value = 0.09840).

A majority of respondents indicated concerns regarding who might enforce adherence to an ELSI-focused computational checklist (68.0%, 51/75), indicated it is unclear how an ELSI-focused computational checklist would be validated or interpreted (60.0%, 45/75), and indicated that the consequences of failing to use an ELSI-focused computational checklist are unclear (50.7%, 38/75). Respondents were divided as to whether the likelihood of adoption was unclear (49.3%, 37/75). Around one-third of respondents (36.0%, 27/75) expressed concern that a checklist approach to ELSI issues would feel like “ethics washing” (i.e., a “rubber stamp” on ethics or a meaningless compliance exercise). Other concerns were less commonly reported by respondents, such as concerns about liability issues (13.3%, 10/75) and concerns about the number of checklists already in existence to consider (16.0%, 12/75).

Generally speaking, roughly half of respondents had a favorable opinion of AI (47.9%, 34/71).

## Discussion

The weak familiarity with either synthetic data or computational checklists reported by respondents to this survey could, at first glance, be viewed as a survey limitation; however, we find this to be a striking result. That individuals employed at AAMC member institutions and holding professional positions reasonably anticipated to involve encounters with AI/ML (either as those likely to use or observe AI/ML in practice or as those responsible for oversight of the use of such AI/ML tools) lack familiarity with the subject matter foci of this survey warrants further examination, as it raises urgent questions about whether biomedical professionals are ready for the widespread integration of AI/ML tools. Another explanation, however, is that with the number of AI/ML tools expanding, it might be increasingly unreasonable to expect everyone to be familiar with each. Training beyond those involved with large-scale NIH-supported efforts (such as the BRIDGE2AI initiative [29]) is needed—particularly if the gap in familiarity that was observed between those self-reporting as AI/ML developers or users (or similar) and those self-reporting as ethicists (or similar) is to be closed.

That said, while we do not view the weak familiarity with synthetic data and computational checklists as a limitation of this survey, there are limitations of the survey that do warrant caution in order to avoid over-interpretation and unsupported generalizations. (1) The survey was not generally administered but, rather, was administered to a target population marked by high educational attainment. (2) The sample of survey respondents contains very little racial and ethnic diversity. (3) While responses were gathered from across the United States, there was limited response from several states (particularly those located in the west region). (4) The completion rate for our exploratory survey was 10.4% (75/722), so there is a possibility of non-response bias. Nevertheless, the results from this survey are useful in providing a foundation upon which further ELSI research can build—whether on the specific areas of focus explored here or on the ample other AI/ML topics that, to date, also have not been given adequate scholarly attention.

### Synthetic data

Regarding synthetic data, our findings show that respondents, regardless of their professional role in biomedicine, generally see the potential value of synthetic data in being one way to address health information privacy concerns as well as concerns about bias. The observed preference for non-synthetic (actual) data over synthetic data, which has also been observed elsewhere by others [12], might simply be an artifact of respondents’ general lack of familiarity with synthetic data as opposed to a deliberate, calculated weighing of relevant factors; however, additional research examining why this preference exists and what factors would influence this preference is needed. Additional research also is needed to better understand the possible relationship and interacting forces between various motivating factors (e.g., pressure to reduce bias in available datasets, pressure to use actual non-synthetic data, or pressure to protect information privacy) and perspectives of and practices with synthetic data.

The broad divide that we observed regarding the potential role of IRBs when synthetic data are used in biomedical contexts seems to be due to several mutually compatible reasons (as suggested by the open-ended explanations we elicited from respondents). Many explanations seemed to reflect a broad interest in respecting or preserving the limitations on IRB authority (i.e., protection of human subjects) and also calibrating IRB oversight to risks involved in the contextualized use of synthetic data (e.g., whether and how synthetic data is being generated and whether already generated synthetic data are being used in particular ways, with particular people, and in particular circumstances).

Transparency is key and needs to be further explored. Currently there is a lack of clear instructions or norms regarding how best to address the generation or use of synthetic data—whether at the time a project involving synthetic data has begun (i.e., disclosure within an institution, such as an IRB or perhaps a data ethics committee) or at the time a project’s outcomes are being reported (i.e., disclosure to others, such as peer reviewers for a journal). The survey responses we collected—combined with insights we gleaned from interviews with key informants reported elsewhere—suggest that synthetic data might be “under the radar” for many biomedical professionals and that guidance is needed to encourage transparency (e.g., how SD were generated, who generated the SD, how well SD performs relative to actual data, etc.) and promote appropriate consideration at different stages of research and/or implementation. Whether this is the job of an IRB is part of that discussion, but it seems apparent that there needs to be a “guardian” of some form to ensure adequate consideration of the ethical issues raised by synthetic data in the biomedical context.

Furthermore, given the lack of familiarity with synthetic data that ethics professionals in biomedical organizations have, as was shown by this survey, there seems to be a serious need for ethnographical work to illuminate the synthetic data practices that are underway so that there is sufficient understanding for a rigorous analysis of the ethical, legal, and social implications to be performed.

### ELSI-focused computational checklists

Regarding the potential utility of ELSI-focused computational checklists to promote responsible biomedical AI/ML, it was a surprising result that our survey respondents had such little familiarity with computational checklists. Computational checklists, such as those used to promote reproducibility [30–31], are already present in data science and healthcare fields—even if not focused on ethical, legal, and social issues per se. Nevertheless, survey respondents (regardless of professional background) expressed a positive initial opinion for using ELSI-focused computational checklists and thought such tools would increase attention to ELSI details. However, the careful design of such checklists and checklist procedures will be critical to prevent “ethics washing”, to encourage thoughtful engagement with social and ethical issues, and to enable nuanced, context-specific disclosures that are shielded from unjustifiably rigid or binary (right/wrong) judgments and shallow criticisms. Our survey did not reveal any obvious consensus among biomedical professionals regarding the items that should be prioritized, although the survey results generally support the notion that priorities should include efforts to reduce bias, promote transparency regarding diversity of actual data used to train the AI model or datasets, preserve privacy, and ensure equitable access to the AI/ML tools developed. While our survey did not examine the underlying motivating or contributing factors influencing prioritization of items for ELSI-focused computational checklists, it would be important to gain such deeper insights as they could ultimately affect user “buy in.” Who would enforce ELSI-focused computational checklists—and how—are important questions that remain open to further discussion as well.

## Conclusion

To conclude, over the past year, we have undertaken several activities to help inform the in-depth efforts of those involved with the ELSI Core for the BRIDGE2AI project and inspire others to dedicate further attention to these matters. Here, we reported the results of our exploratory survey. We separately conducted qualitative research involving key informant interviews, convened a diverse working group of experts intended to help address some of these issues and provide recommendations for policy and practice [32], and hosted a symposium towards responsible biomedical AI [33]. These collective efforts have made it clear that more research is needed, in particular as it relates to preferences for actual versus synthetic data, the role of IRB or similar bodies to provide oversight, and strategies to mitigate some of the challenges our participants highlighted. Moreover, it is clear that increased awareness of SD and computational checklists among biomedical professionals and beyond is needed if one expects to advance the field in a responsible manner. While time and labor intensive, ethnographic work to understand current (and shifting) behaviors, decisions, and practices is critically needed to move beyond surface-level observations and make impactful contributions toward responsible biomedical AI. Such work would provide important substance for consideration by the growing number of groups working to shape policy around the world (e.g., the United Nations AI Advisory Body empaneled to improve global AI governance [34]; the Coalition of Health AI intended to “build a consensus-driven framework” that will produce “guidelines and guardrails” for responsible health AI systems; and the group summoned by the National Academy of Medicine’s Leadership Consortium to develop a code of conduct for healthcare AI [35]).

While synthetic data might currently be a “relatively niche pursuit” [36], the funding opportunities to accelerate AI/ML generally and synthetic data specifically (including but not limited to its use in digital twins and the beginnings of data scientists creating longitudinal synthetic health record datasets [37–38]) and the suggestion by some that synthetic data will ultimately replace actual patient data sets [39] in the near future indicate that additional ELSI research on synthetic data practices and perspectives would be worthwhile alongside technical research. Moreover, we join the voices advocating for human-centered design and the inclusion of diverse perspectives (including specifically through interdisciplinary collaboration) [e.g., [40–42]] to ensure that AI technologies are developed, used, and refined in ways that both advance health equity and facilitate ethical reflection. We also underscore the need for further ELSI research offering practical insights to help shape emerging codes of ethics and conduct for healthcare AI [43–44] as well as emerging sociotechnical standards such as those supported by IEEE working groups [45].

## Supporting information

S1 Appendix Copy of instrument survey preview. (PDF)

S1 Data Survey data file with analysis. (XLSX)
