# Supplementary material for: Synthetic data and ELSI-focused computational checklists—A survey of biomedical professionals’ views
Source: PLOS Digit Health. 2024 Nov 20;3(11):e0000666. doi: 10.1371/journal.pdig.0000666 (PMC11578457; doi:10.1371/journal.pdig.0000666)
Supplement: S1 Appendix — (PDF) [file pdig.0000666.s001.pdf]

Introduction

.

Study Title: Exploring the Ethics of Synthetic Data & Artificial Intelligence

Researchers: This study is led at Penn State University (PSU) by Dr. Jennifer K. Wagner (Principal Investigator) in collaboration with Dr. Laura Y. Cabrera, Ms. Sara Gerke, Dr. Daniel Susser at PSU and Dr. Alex Bui and Dr. Anders Garlid at UCLA.

Purpose: We are conducting research to explore the ethics of synthetic data and ethics-focused checklists to promote responsible use of artificial intelligence in biomedical contexts.

Participation: We are asking adults (individuals at least 18 years of age) whom we have identified as having relevant expertise to participate in this survey. The survey will take about 15-20 minutes to complete. Your participation in this survey is voluntary. You may choose not to answer any questions and may end the survey at any time.

Benefits and Risks: There are no direct benefits to you for participating in this survey. The risks of this research are no more than those ordinarily encountered in daily life when you share information online. The topics covered in the survey are not inherently upsetting or sensitive and do not involve matters that, if disclosed, would risk damage to your reputation, employment, financial standing, or liability. This survey is anonymous. We are not collecting identifying information and are not linking responses to your identity. We may share survey response data with others, including when we publish the results of our study.

Compensation: Individuals completing the survey will be offered a $25 e-gift card through TangoCard. Any identifying information that you provide to obtain the gift card will be kept confidential by the study team.

Sponsors: This study is funded in part by the National Institutes of Health (NIH OD and NIBIB Grant No. 3R01EB027650-03S1) and by the Rock Ethics Institute at PSU.

Questions: If you have questions about this survey, you may email Jennifer K. Wagner at jkw131@psu.edu or call (814) 865-2952 and mention PSU IRB # STUDY00020918.

Electronic Consent: If you would like to participate, please click the button below to agree to participate and start the survey.

○ Yes, I agree to participate.

Basic Questions About You

Q1. In which age group do you belong?

- ○ 18 to 25 years old
- ○ 26 to 35 years old
- ○ 36 to 45 years old
- ○ 46 to 55 years old
- ○ 56 to 65 years old
- ○ 66 to 75 years old
- ○ 76 years and older

Q2. What terms best express hold you describe your gender identity? Select all that apply.

- ☐ Male
- ☐ Female
- ☐ Non-binary
- ☐ Transgender
- ☐ None of these describe me. I identify as:

Q3. Which categories describe you? Select all that apply.

- ☐ American Indian or Alaska Native
- ☐ Asian
- ☐ Black, African American, or African
- ☐ Hispanic, Latino, or Spanish
- ☐ Middle Eastern or North African
- ☐ Native Hawaiian or other Pacific Islander
- ☐ White
- ☐ None of these fully describe me. I identify as:

Q4. What is the highest grade or year of school you completed?

- ○ Grade 11 or below
- ○ Grade 12 or GED (high school graduate)

- 1 to 3 years after high school (some college, associate's degree, or technical school)
- College 4 years or more (college graduate)
- Advanced degree (master's, doctorate, etc.)

Q5. Which of the following best describes your current employer?

- Medical School
- Teaching Hospital or Healthcare System
- College or University
- None of these. I would describe my employer as:

Q6. Which option best describes your current professional role?

- Data scientist or engineer; informaticist or informatician; AI/ML developer or user; information, technology, or content officer; or similar
- Ethics researcher; ethicist; ethics or compliance officer; Institutional Review Board (IRB) member, director, or administrator; or similar
- Neither of these. I would describe my professional role as:

Q7. How much experience do you have in your current professional role?

- 0-5 years
- 6-10 years
- more than 10 years

Q8. Where in the United States do you currently work?

About Synthetic Data

Q9. How familiar are you with synthetic data?

- Not at all familiar
- Slightly familiar
- Moderately familiar

- Very familiar
- Extremely familiar

Q10. Have you encountered synthetic data in your work?

- No
- Not Sure
- Yes

Q11. What is your general opinion of the use of synthetic data in biomedical contexts?

- Very favorable
- Favorable
- Neither favorable nor unfavorable
- Unfavorable
- Very unfavorable

Q12. Which of the following statements best describes your perspective on the relationship between synthetic data and real world data in biomedical contexts?

- I generally do not have a preference between synthetic data and real world data
- I generally prefer synthetic data over real world data
- I generally prefer real world data over synthetic data

Q13. How familiar are you with the method(s) used to generate synthetic data?

- Not at all familiar
- Slightly familiar
- Moderately familiar
- Very familiar
- Extremely familiar

Q14. To what extent can synthetic data address health information privacy concerns?

- Not at all
- Somewhat
- Mostly
- Completely

Q15. To what extent can synthetic data address bias concerns?

- Not at all

- Somewhat
- Mostly
- Completely

Q16. Which of the following statements best reflects your perspective of Institutional Review Boards' (IRBs') role in oversight of the use of synthetic data in biomedical contexts?

- IRBs NEVER should oversee the use of synthetic data in biomedical contexts.
- IRBs ALWAYS should oversee the use of synthetic data in biomedical contexts.
- IRBs SOMETIMES should oversee the use of synthetic data in biomedical contexts.
- I am not sure whether IRBs should oversee the use of synthetic data in biomedical contexts.

Q17. Thinking about your answer to the previous question, why do you have that perspective of possible IRB oversight of the use of synthetic data in biomedical contexts?

Q18. With which of the following items do you agree? Select all that apply.

- [ ] Synthetic data might disincentivize researchers from engaging individuals, groups, and communities who are underrepresented
- [ ] Synthetic data might cause problems with accountability
- [ ] The quality of synthetic data might be difficult to determine
- [ ] Synthetic data might be used to evade human subjects research protections
- [ ] Researchers using synthetic data might not disclose that their studies rely on synthetic data
- [ ] Synthetic data might exacerbate data inequities
- [ ] Synthetic data uses might disincentivize researchers from returning study findings to individuals, groups, and communities
- [ ] I have other concerns: (please describe)

Ethics-Focused Computational Checklists for AI/ML

Q19. Standards for documenting and communicating details about Artificial Intelligence (AI) and Machine Learning (ML) objects (such as datasets and models) are of increasing

importance for promoting reproducibility and responsible re-use. How familiar are you with computational checklists for AI developers to describe models in standardized ways?

- ○ Not at all familiar
- ○ Slightly familiar
- ○ Moderately familiar
- ○ Very familiar
- ○ Extremely familiar

Q20. Computational checklists for AI developers are intended to allow common elements or features to be disclosed so that predictive models can be evaluated and compared consistently with one another. Which option best reflects your initial reaction to the idea of creating and using computational checklists for ethics-related aspects of AI models and datasets?

- ○ I STRONGLY OPPOSE the idea of ethics-focused computational checklists.
- ○ I OPPOSE the idea of ethics-focused computational checklists.
- ○ I SUPPORT the idea of ethics-focused computational checklists.
- ○ I STRONGLY SUPPORT the idea of ethics-focused computational checklists.

Q21. Thinking about your answer to the previous question, what is the main reason for your initial reaction to the idea of ethics-focused computational checklists for AI developers?

[ ]

Q22. Type 1, 2, and 3 to rank the three most important ethics-focused aspects of AI models or datasets that should be documented and communicated to promote ethical and trustworthy AI. (In other words, what are the top three ethics-related details that everyone should know about regarding a specific AI model or dataset before using it?)

[ ] Details regarding patient and community engagement during the development of the AI model or dataset

[ ] Diversity of expertise among members of the team who developed the AI model or dataset

[ ] Steps taken to reduce bias in the AI model or dataset

[ ] Sources of funding that supported the development of the AI model or dataset

[ ] Reputation of the institution or organization that developed the AI model or dataset

[ ] Details regarding any conditions or restrictions on future uses of the AI model or dataset

[ ] Steps taken to preserve information privacy and security in the design of the AI model or dataset

[ ] Steps taken to ensure that access to the AI model or dataset is equitable

- [ ] Informed consent documentation
- [ ] Characteristics of the individuals or groups used to train the AI model or dataset

Q23. There is concern about overburdening AI developers and researchers with documentation and other regulatory obligations. Which option best reflects your initial reaction to the idea of creating and using automated processes to validate disclosures of ethics-focused features of AI models and datasets?

- ○ I STRONGLY SUPPORT the idea of automation of ethical considerations for AI models and datasets
- ○ I SUPPORT the idea of automation of ethical considerations for AI models and datasets
- ○ I OPPOSE the idea of automation of ethical considerations of AI models and datasets
- ○ I STRONGLY OPPOSE the idea of automation of ethical considerations for AI models and datasets

Q24. How (if at all) do you think the use of ethics-focused computational checklists would affect the quality of attention given to ethics-related matters when developing and using AI models and datasets?

- ○ Ethics-focused computational checklists would INCREASE the quality of attention given to ethical dimensions of AI models and datasets.
- ○ Ethics-focused computational checklists would HAVE NO EFFECT ON the quality of attention given to ethical dimensions of AI models and datasets.
- ○ Ethics-focused computational checklists would DECREASE the quality of attention given to ethical dimensions of AI models and datasets.

Q25. With which of the following statements about ethics-focused computational checklists for AI do you agree? Select all that apply.

- [ ] An ethics-focused computational checklist feels like "ethics washing" (e.g., a "rubber stamp" on ethics or a meaningless compliance exercise)
- [ ] An ethics-focused computational checklist would oversimplify the issues
- [ ] The likelihood of adoption is unclear
- [ ] There are already too many computational checklists to consider
- [ ] The consequences of failing to use an ethics-focused computational checklist are unclear
- [ ] There are questions about who might enforce adherence to an ethics-focused computational checklist
- [ ] It is unclear how an ethics-focused computational checklist would be validated or interpreted
- [ ] An ethics-focused computational checklist raises liability concerns

- [ ] I have other concerns: (please describe)

Q26. Which option best describes how you would feel upon learning that ethics-focused computational checklists for AI models and datasets in biomedical contexts are required for federally-funded research?

- ○ Excited, enthusiastic, or hopeful
- ○ Curious or watchful
- ○ Indifferent or unmoved
- ○ Worried, nervous, or uneasy
- ○ Angry or betrayed

About AI Ethics Generally

Q27. What is your general opinion of the use of AI in society?

- ○ Very unfavorable
- ○ Unfavorable
- ○ Neither favorable nor unfavorable
- ○ Favorable
- ○ Very favorable

Q28. Are you aware that a Blueprint for an AI Bill of Rights was recently released by the Office of Science and Technology Policy (OSTP)?

- ○ Yes
- ○ Not Sure
- ○ No

Q29. To what extent is the development and implementation of an AI Bill of Rights a priority to you personally?

- ○ Not a priority
- ○ Low priority
- ○ Medium priority
- ○ High priority

○ Essential

Q30. Is there anything else you want to share regarding synthetic data, ethics-focused computational checklists, or how to promote responsible use of AI in biomedical contexts?

Powered by Qualtrics
